# Inflammatory Biomarkers, Death, and Recurrent Nonfatal Coronary Events After an Acute Coronary Syndrome in the MIRACL Study

**DOI:** 10.1161/JAHA.112.003103

**Published:** 2013-02-22

**Authors:** Payman Zamani, Gregory G. Schwartz, Anders G. Olsson, Nader Rifai, Weihang Bao, Peter Libby, Peter Ganz, Scott Kinlay, for the Myocardial Ischemia Reduction with Aggressive Cholesterol Lowering (MIRACL) Study Investigators

**Affiliations:** 1 Cardiovascular Division, University of California, San Diego, CA (P.Z.); 2 Cardiology Division, Veterans Affairs Medical Center and University of Colorado, Denver, CO (G.G.S.); 3 Faculty of Health Sciences, University of Linköping, Linköping, Sweden (A.G.O.); 4 Children's Hospital Boston and Harvard Medical School, Boston, MA (N.R.); 5 Pfizer Pharmaceuticals Group, New York, NY (W.B.); 6 Cardiovascular Division, Brigham and Women's Hospital and Harvard Medical School, Boston, MA (P.L., S.K.); 7 Cardiovascular Division, San Francisco General Hospital and University of California, San Francisco, CA (P.G.); 8 Cardiovascular Division, Veterans Affairs Boston Healthcare System, West Roxbury, Harvard Medical School, Boston, MA (S.K.)

**Keywords:** acute coronary syndromes, biomarkers, CRP, death, nonfatal events, risk

## Abstract

**Background:**

In acute coronary syndromes, C‐reactive protein (CRP) strongly relates to subsequent death, but surprisingly not to recurrent myocardial infarction. Other biomarkers may reflect different processes related to these outcomes. We assessed 8 inflammatory and vascular biomarkers and the risk of death and recurrent nonfatal cardiovascular events in the 16 weeks after an acute coronary syndrome.

**Methods and Results:**

We measured blood concentrations of CRP, serum amyloid A (SAA), interleukin‐6 (IL‐6), soluble intercellular adhesion molecule (ICAM), soluble vascular cell adhesion molecule (VCAM), E‐selectin, P‐selectin, and tissue plasminogen activator antigen (tPA) 24 to 96 hours after presentation with acute coronary syndrome in 2925 subjects participating in a multicenter study. Biomarkers were related to the risk of death, and recurrent nonfatal acute coronary syndromes (myocardial infarction or unstable angina) over 16 weeks using Cox proportional hazard models. On univariate analyses, baseline CRP (*P*=0.006), SAA (*P*=0.012), and IL‐6 (*P*<0.001) were related to death, but not to recurrent nonfatal acute coronary syndromes. VCAM and tPA related to the risk of death (*P*<0.001, *P*=0.021, respectively) and to nonfatal acute coronary syndromes (*P*=0.021, *P*=0.049, respectively). Adjusting for significant covariates reduced the strength of the associations; however, CRP and SAA continued to relate to death.

**Conclusions:**

In acute coronary syndromes, the CRP inflammatory axis relates to the risk of death and may reflect myocardial injury. VCAM and tPA may have greater specificity for processes reflecting inflammation and thrombosis in the epicardial arteries, which determine recurrent coronary events.

## Introduction

Inflammation and thrombosis are key components of both myocardial necrosis and disrupted coronary plaques in acute coronary syndromes.^1^ Shortly after these events, plasma concentration of the hepatic acute phase reactant C‐reactive protein (CRP) increases several fold.^2–4^ Although high concentrations of CRP relate to subsequent mortality over several months after an acute coronary syndrome, CRP is not related to recurrent myocardial infarction.^5–13^ Some authors speculate that in the acute coronary syndrome setting, elevated CRP predominantly reflects the extent of myocardial injury, which determines early mortality, rather than inflammation in the coronary arterial system, which may drive recurrent nonfatal coronary events.^14^

Support for this hypothesis could come from several previously unexplored lines of evidence. These include whether there are similar relationships between mortality and recurrent nonfatal acute coronary syndromes with other biomarkers that either stimulate CRP (eg, interleukin‐6) or track closely with it (eg, SAA) and whether other markers that are more closely linked to arterial inflammation and thrombosis have different relationships to these clinical end points. Inflammatory biomarkers, which reflect processes more specific to endothelial and coronary artery thrombosis, include soluble selectins, cellular adhesion molecules, and endogenous tissue plasminogen activator.

The Myocardial Ischemia Reduction with Aggressive Cholesterol Lowering (MIRACL) study provided an opportunity to measure multiple biomarkers simultaneously for this purpose. We aimed to assess how various baseline biomarkers of inflammation, sampled 24 to 96 hours after hospital admission for an acute coronary syndrome, associated with different clinical outcomes up to 4 months after an acute coronary syndrome.

## Methods

### Study Population

The MIRACL study enrolled 3086 subjects admitted with unstable angina or non‐Q‐wave myocardial infarction to 122 hospitals in Europe, North America, South Africa, and Australia.^15^ These diagnoses required chest discomfort lasting ≥15 minutes within the 24 hours preceding hospitalization and representing a change in the usual pattern of angina. The diagnosis of unstable angina required new or dynamic ST‐wave or T‐wave changes in ≥2 contiguous ECG leads or a new wall‐motion or myocardial perfusion abnormality. The diagnosis of myocardial infarction required elevation of serum creatine kinase, its MB fraction, or troponin to a level exceeding 2 times the upper limit of normal. All patients provided informed consent. The protocol was approved by local institutional review boards.

Subjects were excluded if coronary revascularization was planned or anticipated. Subjects were randomized 24 to 96 hours after admission to atorvastatin 80 mg daily or matching placebo for 16 weeks. The combined primary end point for the MIRACL study was a recurrent ischemic event, defined as death, nonfatal myocardial infarction, recurrent unstable angina, or resuscitated cardiac arrest. The end points for this analysis were all‐cause death and recurrent nonfatal acute coronary syndromes (ie, acute myocardial infarction and recurrent unstable angina requiring emergency hospitalization and with objective evidence of myocardial ischemia as defined above). Patients with recurrent nonfatal acute coronary syndromes who subsequently died during the 16‐week follow‐up were counted as deaths only.

### Measurement of Biomarkers

Blood was collected into serum and EDTA tubes 24 to 96 hours after hospital admission for an acute coronary syndrome to measure baseline levels of biomarkers. The tubes were centrifuged on site, and the serum or plasma was shipped to the core laboratory for storage at −70°C. Samples were then sent to the inflammatory markers core laboratory, where they were measured in batches.^16–18^ High‐sensitivity CRP and serum amyloid A (SAA) were measured by high‐sensitivity immunonephelometry (Dade Behring, Deerfield, IL). Interleukin‐6 (IL‐6), soluble intercellular adhesion molecule (ICAM), soluble vascular cell adhesion molecule (VCAM), soluble E‐selectin, and soluble P‐selectin were measured by ELISA (R&D Systems, Minneapolis, MN), and endogenous tissue plasminogen activator antigen (tPA) was measured by ELISA (American Diagnostica, Greenwich, CT). Troponin I was measured using an ACS:180 Chemiluminescence cTNI Immunoassay (Bayer Diagnostics, Tarrytown, NY). The coefficients of variation for reproducibility of the assays were excellent: CRP, 4.5% at 12.6 mg/L and 5.2% at 48 mg/L; SAA, 6.2% at 14.8 mg/L; IL‐6, 11.6% at 1.1 pg/mL and 7.0% at 4.7 pg/mL; ICAM, 7.4% at 117 ng/mL and 6.1% at 453 ng/mL; VCAM, 8.5% at 24.9 ng/mL and 8.9% at 49.6 ng/mL; E‐selectin, 8.8% at 20.4 ng/mL and 7.4% at 115 ng/mL; P‐selectin, 9.9% at 94 ng/mL and 7.9% at 730 ng/mL; tPA, 5.5% at 6.0 ng/mL and 4.9% at 15.0 ng/mL.

### Subgroup With Known Baseline Ejection Fraction

In 910 subjects, baseline left ventricular ejection fraction (LVEF) was documented from echocardiography, angiography, or a nuclear medicine study. In this subgroup we explored the relationship between abnormal LVEF (defined as <55%) and the biomarkers and clinical outcomes.

### Statistics

This report focuses on the subjects in the MIRACL study who had baseline levels of inflammatory markers measured prior to randomization, including 2925 (95% of all MIRACL subjects) for CRP, 2852 (92%) for IL‐6, 2923 (95%) for SAA, 2858 (93%) for ICAM, 2860 (93%) for VCAM, 2633 (85%) for E‐selectin, 2858 (93%) for P‐selectin, and 2780 (90%) for tPA. As the distributions of inflammatory markers were skewed, biomarkers are described using median and interquartile ranges (25% to 75%) and models used nonparametric quartile analysis. We used chi‐square tests for categorical variables and Kruskall–Wallis tests for continuous variables to compare baseline variables and the continuous distribution of biomarkers among subjects who died, had recurrent nonfatal acute coronary syndromes, or had no adverse events on follow‐up. Cox proportional hazards models were used to assess the relative risk (hazard ratio) for events during the 16 weeks of the study over quartiles of biomarkers, with the lowest quartile as the reference category. A linear trend test was also included to assess whether the risk of clinical outcome was monotonically increasing or decreasing over quartiles of biomarker level. As there were no significant treatment‐by‐biomarker interaction effects in the models, results for both treatment groups (atorvastatin and placebo) were combined with treatment as a covariate. Multivariable models were used, adjusting for treatment assignment and other significant covariates related to recurrent events (see Table 1). Because of the exploratory nature of this study, 2‐sided nominal (uncorrected for multiple testing) *P* values are reported for all statistical comparisons based on our prespecified analysis plan.

**Table 1.** Baseline Characteristics of Subjects Who Died, Had a Nonfatal ACS, or Did Not Have an Event Over the 16 Weeks of the Study (*P* Value From Kruskall–Wallis)

	Death (n=127)	Nonfatal ACS (n=348)	No Event (n=2450)	*P* Value
Age, mean (SD)	75 (10)	68 (12)	65 (12)	<0.001
Male (%)	81 (64)	232 (67)	1591 (65)	0.81
Past MI (%)	56 (44)	114 (33)	558 (23)	<0.001
Prior coronary revascularization	20 (16)	43 (12)	238 (10)	0.04
Hypertension (%)	82 (65)	184 (53)	1326 (54)	0.058
Current smoker (%)	14 (11)	81 (23)	723 (30)	<0.001
Diabetes (%)	44 (35)	91 (26)	530 (22)	<0.001
Body mass index (kg/m^2^), mean (SD)	26.9 (8.5)	27.4 (4.4)	27.6 (5.2)	0.052
Admission to randomization (h), mean (SD)	68 (45)	63 (23)	63 (23)	0.45
LVEF (%), median (25%, 75%)*	45 (33, 55)	50 (42, 61)	56 (47, 65)	<0.001
Total cholesterol (mg/dL), mean (SD)	200 (48)	204 (41)	206 (37)	0.12
HDL (mg/dL), mean (SD)	45 (13)	45 (11)	47 (13)	0.002
LDL (mg/dL), mean (SD)	122 (40)	123 (34)	124 (33)	0.61
Triglycerides (mg/dL), mean (SD)	166 (69)	186 (83)	183 (92)	0.09
Troponin I, ng/mL (%)	64 (50)	140 (40)	1042 (43)	0.10
CRP (mg/L), median (25%, 75%)	20.1 (6.0, 52.9)	10.2 (4.5, 33.1)	10.2 (4.2, 35.0)	0.005
IL‐6 (pg/mL), median (25%, 75%)	10.8 (5.5, 20.8)	7.1 (3.7, 14.1)	6.7 (3.7, 14.6)	<0.001
SAA (mg/dL), median (25%, 75%)	2.5 (0.7, 19.7)	1.4 (0.5, 8.6)	1.6 (0.6, 8.2)	0.02
VCAM (ng/L), median (25%, 75%)	959 (674, 1231)	870 (656, 1069)	808 (619, 1020)	<0.001
ICAM (ng/L), median (25%, 75%)	307 (259, 366)	302 (263, 366)	299 (252, 358)	0.17
P‐Selectin (ng/L), median (25%, 75%)	65 (46, 96)	59 (42, 90)	59 (41, 93)	0.56
E‐Selectin (ng/L) median (25%, 75%)	47 (34, 63)	47 (33, 62)	48 (35, 62)	0.63
tPA (ng/L), median (25%, 75%)	15.1 (10.7, 24.3)	14.6 (9.6, 21.5)	13.5 (8.5, 20.3)	0.003

ACS indicates acute coronary syndrome; MI, myocardial infarction; LVEF, left ventricular ejection fraction; HDL, high‐density lipoprotein; LDL, low‐density lipoprotein; CRP, C‐reactive protein; IL‐6, interleukin‐6; SAA, serum amyloid A; VCAM, vascular cell adhesion molecule; ICAM, intercellular adhesion molecule; tPA, tissue plasminogen activator antigen.

* LVEF measured in 910 subjects.

## Results

Of the 2925 subjects with CRP measured in the MIRACL study, 127 (4%) died, 205 (7%) had recurrent myocardial infarction, and 215 (7%) had recurrent unstable angina with objective evidence of ischemia over the 16 weeks of the study.

Table 1 compares baseline descriptive data on subjects who had an event (death, nonfatal MI, or unstable angina) and those who remained event free. Patients who had an event were older (*P*<0.001); were more likely to have a history of past myocardial infarct (*P*<0.001), prior revascularization (*P*=0.04), or diabetes (*P*<0.001); were less likely to be current smokers (*P*<0.001); and had lower high‐density lipoprotein (HDL) cholesterol (*P*=0.002) and lower LVEF (*P*<0.001). Their baseline concentrations of CRP, IL‐6, SAA, VCAM, and tPA were significantly higher, and ICAM tended to be higher (Table 1).

In the following univariate and multivariable models, atorvastatin associated with a 3% to 9% reduction in the risk of death and a 19% to 22% reduction in the risk of recurrent nonfatal acute coronary syndromes. However, as there were no significant treatment‐by‐biomarker interactions, we combined the atorvastatin and placebo groups and included treatment as a covariate. Also, we chose to assess the relationship of biomarkers to outcomes using test‐of‐trend statistics, which reflect the continuous relationship of biomarkers to outcomes. However, our multivariable models also showed hazard ratios for each quartile compared with the reference (first) quartile.

### CRP, IL‐6, and SAA

**Figure 1.**
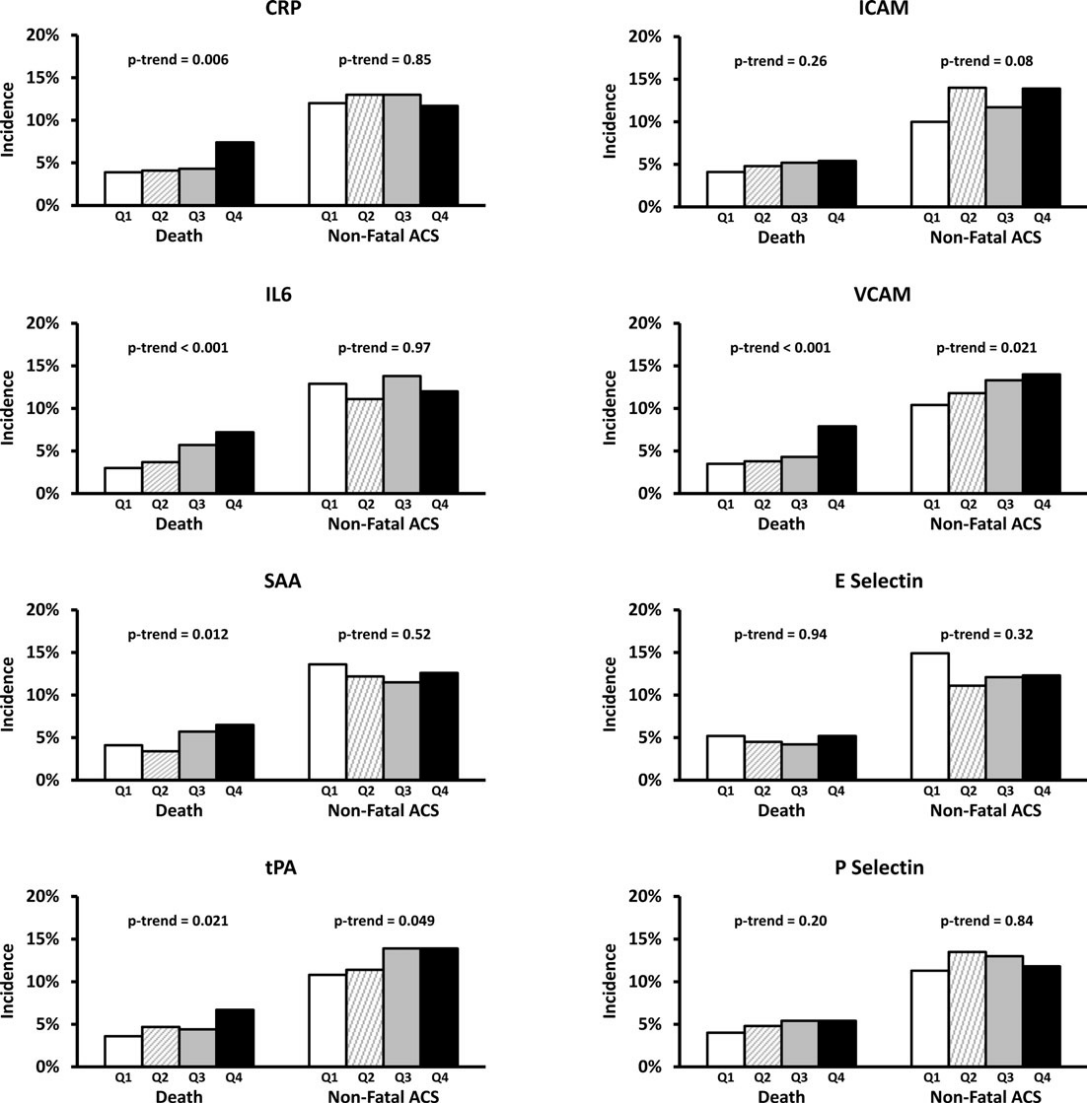
Incidence rates and test‐of‐trend for the risk of death and recurrent nonfatal acute coronary syndromes (ACS) of myocardial infarction or unstable angina, associated with quartiles of each biomarker. CRP indicates C‐reactive protein; ICAM, intercellular adhesion molecule; IL‐6, interleukin‐6; VCAM, vascular cell adhesion molecule; SAA, serum amyloid A; tPA, tissue plasminogen activator antigen.

Univariate analyses showed strong correlations between death and increasing quartiles of baseline CRP, IL‐6, and SAA (Figure 1). Interestingly, these CRP axis biomarkers did not correlate with recurrent nonfatal acute coronary events (Figure 1).

### VCAM, tPA, and Other Biomarkers

In univariate analyses, baseline quartiles of VCAM and tPA concentrations associated with death as well as with recurrent nonfatal coronary events (Figure 1). None of the other biomarkers bore any relationship either to death or to recurrent nonfatal acute coronary syndromes (Figure 1).

### Biomarkers and Risk After Adjustment for Other Covariates

Adjustment for covariates in multivariable models attenuated the statistically significant hazard ratios. For example, for fatal events, multivariable adjustment reduced the hazard ratio for the fourth compared with the first quartile for CRP (from 1.92 to 1.69), IL‐6 (from 2.40 to 1.61), SAA (from 1.61 to 1.41), VCAM (from 2.36 to 1.12), and tPA (from 1.90 to 1.27). Similarly, multivariable adjustment attenuated the hazard ratios for nonfatal acute coronary syndromes for the fourth compared with the first quartile for VCAM (from 1.41 to 1.12) and tPA (1.31 to 1.20). In the multivariable models, CRP and SAA associated with a higher risk of death (*P*=0.034 and *P*=0.049, respectively; Table 2). However, no other biomarkers were related to death, and none of the other biomarkers were related to nonfatal acute coronary syndromes in multivariable models (Table 2).

**Table 2.** Hazard Ratios for Quartiles of Biomarkers at Baseline and Death and Recurrent Nonfatal ACS of Myocardial Infarction or Unstable Angina

	Quartile 1, Reference	Quartile 2, HR (95% CI)	Quartile 3, HR (95% CI)	Quartile 4, HR (95% CI)	Test‐of‐Trend
CRP					
Death	1.00	0.97 (0.54 to 1.75)	1.08 (0.61 to 1.91)	1.69 (0.99 to 2.90)	0.034
Nonfatal ACS	1.00	1.06 (0.78 to 1.44)	1.09 (0.80 to 1.49)	0.98 (0.70 to 1.36)	0.941
IL‐6					
Death	1.00	1.05 (0.56 to 1.98)	1.29 (0.71 to 2.35)	1.61 (0.90 to 2.90)	0.060
Nonfatal ACS	1.00	0.82 (0.60 to 1.13)	0.94 (0.69 to 1.27)	0.84 (0.61 to 1.16)	0.439
SAA					
Death	1.00	0.66 (0.36 to 1.21)	1.14 (0.66 to 1.97)	1.41 (0.82 to 2.43)	0.045
Nonfatal ACS	1.00	0.87 (0.65 to 1.18)	0.85 (0.62 to 1.16)	0.96 (0.70 to 1.33)	0.783
ICAM					
Death	1.00	1.21 (0.69 to 2.10)	1.26 (0.73 to 2.19)	1.53 (0.89 to 2.62)	0.131
Nonfatal ACS	1.00	1.41 (1.03 to 1.94)	1.18 (0.84 to 1.65)	1.48 (1.07 to 2.05)	0.064
VCAM					
Death	1.00	0.83 (0.45 to 1.52)	0.74 (0.40 to 1.34)	1.12 (0.65 to 1.94)	0.484
Nonfatal ACS	1.00	1.09 (0.79 to 1.51)	1.11 (0.80 to 1.54)	1.12 (0.81 to 1.55)	0.531
E‐selectin					
Death	1.00	1.01 (0.59 to 1.73)	0.92 (0.52 to 1.62)	1.26 (0.74 to 2.13)	0.486
Nonfatal ACS	1.00	0.77 (0.56 to 1.06)	0.85 (0.62 to 1.16)	0.87 (0.64 to 1.20)	0.489
P‐selectin					
Death	1.00	1.40 (0.79 to 2.45)	1.69 (0.98 to 2.93)	1.71 (0.99 to 2.95)	0.043
Nonfatal ACS	1.00	1.30 (0.95 to 1.77)	1.29 (0.94 to 1.77)	1.13 (0.82 to 1.56)	0.508
tPA					
Death	1.00	1.17 (0.67 to 2.03)	0.83 (0.46 to 1.48)	1.27 (0.75 to 2.16)	0.574
Nonfatal ACS	1.00	1.01 (0.73 to 1.41)	1.16 (0.84 to 1.59)	1.20 (0.88 to 1.65)	0.178

Hazard ratios are adjusted by treatment group, age, past myocardial infarction, prior coronary revascularization, diabetes mellitus, current smoking, HDL cholesterol, and positive troponin. ACS indicates acute coronary syndrome; CRP, C‐reactive protein; IL‐6, interleukin‐6; SAA, serum amyloid A; ICAM, intercellular adhesion molecule; VCAM, vascular cell adhesion molecule; tPA, tissue plasminogen activator antigen.

### Subgroup With Known Baseline Ejection Fraction

Among the 910 subjects with LVEF assessed at baseline, 402 (44%) had an abnormal LVEF of <55%. Compared with normal LVEF, abnormal LVEF associated with higher median CRP (9.2 versus 14.6 mg/L, *P*<0.0001), IL‐6 (5.9 versus 8.6 pg/L, *P*<0.0001), SAA (1.3 versus 2.4 mg/L, *P*<0.0001), and VCAM (765 versus 857 ng/L, *P*=0.0027), but was unrelated to the other baseline biomarkers. Abnormal LVEF was also related to death (HR 3.14, 95% CI 1.56 to 6.34, *P*=0.0014) and recurrent nonfatal acute coronary syndromes (HR 1.55, 95% CI 1.05 to 2.30, *P*=0.03).

## Discussion

Our analysis of the MIRACL study provides several novel findings relating biomarkers to the early risk of death or recurrent ischemic coronary events after an acute non‐ST‐segment elevation coronary syndrome. We have extended the previously described association between CRP and death to other members of the CRP axis, namely, SAA and IL‐6. We have also extended the previously described absence of a relationship between CRP and nonfatal events to SAA and IL‐6. In contrast, our study found that VCAM and tPA, markers of endothelial activation and thrombosis, were related both to mortality and to recurrent acute coronary syndromes. Recurrent myocardial infarction and unstable angina share a similar biology (plaque disruption and thrombosis) and justify combining these end points. A major strength of our study was the simultaneous comparison of multiple biomarkers, beyond CRP, within the same cohort with a large sample size.

### CRP, SAA, and IL‐6

IL‐6 is released in relatively small concentrations into the bloodstream from numerous cell types associated with inflamed plaques.^19^ In the liver, this molecule promotes the production of CRP and SAA, which are released into the bloodstream in far greater concentrations.^19^ As IL‐6 is the primary driver of CRP and SAA production, these biomarkers align in a common pathway. Our study demonstrates that they relate to cardiovascular risk in a similar manner. A novel feature of this analysis was the evaluation of several biomarkers in this inflammatory axis.

In other studies of acute coronary syndromes, higher CRP at presentation relates to death in the short term^11,13,20–21^ and in follow‐up exceeding 6 months after the initial event.^5,20,22–24^ However, CRP fails to relate consistently to the wider range of cardiovascular events including recurrent myocardial infarction.^6,13^ With longer follow‐up after an acute coronary syndrome or in patients with chronic coronary heart disease, CRP does relate both to fatal and nonfatal MI^25–26^and reflects known relationships between low‐grade inflammation in chronic coronary artery disease and the long‐term risk of cardiovascular events. Our study provides further evidence that levels of CRP and its related biomarkers SAA and IL‐6 do not relate to the short‐term risk of nonfatal cardiovascular outcomes, but do relate to death.

Why this inflammatory axis relates only to death but not to other cardiovascular end points remains unclear. One hypothesis is that higher CRP in the setting of an acute coronary syndrome reflects a greater burden of myocardial ischemia and tissue injury.^14^ Most studies of acute coronary syndromes, including MIRACL, support this concept, as subjects with higher troponins also have higher concentrations of CRP.^11,16,22,27^ Some earlier reports did not find a relationship between CRP and troponin, but this disparity may have been a result of the use of less sensitive troponin assays.^5,28^

Our subgroup analysis of patients with known LVEF at baseline also supports this concept. Abnormal LVEF associated with higher concentrations of CRP, SAA, and IL6 and to a higher risk of death. This suggests that these biomarkers reflect greater myocardial damage or less reserve, which drives the strong relationship of these biomarkers to death.

In other smaller reports, higher CRP concentrations shortly after an acute coronary syndrome related to more severe wall‐motion abnormalities on echocardiography, a higher incidence of heart failure,^21^ and a higher risk of cardiac rupture or aneurysm formation after a Q‐wave MI.^29^ Our study shows consistent relationships to short‐term outcomes across this inflammatory axis and suggests that CRP, SAA, and IL‐6 reflect a more severe and complicated consequence of acute coronary syndrome that relates to death.

### Cellular Adhesion Molecules, Selectins, and tPA

In contrast to the CRP axis, VCAM and tPA related both to death and to recurrent nonfatal acute coronary syndromes of myocardial infarction or unstable angina. Activated endothelial cells express E‐selectin, VCAM, and ICAM, adhesion molecules implicated in leukocyte recruitment and migration into the vessel wall.^30–31^ P‐selectin and tPA also reflect platelet activation and the balance of fibrin formation and fibrinolysis.^17,32^ Thus, these markers relate more directly to biological mechanisms that promote arterial thrombus and subsequent fatal and nonfatal cardiovascular events.

### Biomarkers and Risk After Multivariable Adjustment

After adjusting for known significant covariates, most of the hazard ratios for biomarkers were lower, and some no longer remained statistically significant. This was expected, as most biomarkers reflect inflammatory and thrombotic processes that are exacerbated by covariates such as age, HDL cholesterol, diabetes, and degree of myocardial injury (troponin). In other words, the biomarkers assess the causal pathway through which upstream risk factors (our covariates) exert their adverse effect on outcomes; any relationship of biomarkers to outcome will be diluted by models that include more proximal factors in these processes. The implication of this attenuation in risk is that the biomarkers provide valuable insights into the mechanisms relating clinical outcomes to myocardial injury, arterial inflammation, and thrombosis, but may not substantially enhance clinical risk prediction models.

### Limitations

The prespecified analysis plan made no correction for multiple comparisons because of the exploratory nature of this analysis, and the data on VCAM and tPA are hypothesis generating and require confirmation in a separate study. As many significance tests are reported, nominal *P* values should be interpreted with caution. The biomarkers in our study may have different long‐term relationships to outcomes beyond the 16 weeks of this study. Also, we could not assess the affect of sampling biomarkers at different times after the onset of acute coronary syndrome. However, peak levels of CRP and SAA occur 1 to 3 days after an acute inflammatory insult, which was within the time frame of the baseline biomarkers.^19^

## Conclusions

Our analysis of the MIRACL study shows a strong relationship between early levels of CRP and the closely related markers of IL‐6 and SAA with death following an acute coronary syndrome. These biomarkers are not associated with recurrent nonfatal coronary events. In contrast, early levels of VCAM and tPA are related to both mortality and recurrent nonfatal coronary syndromes and may have greater specificity for processes reflecting inflammation and thrombosis in the epicardial arteries, which determine recurrent coronary events.
